# A novel phospholipase A2 is a core component of the typhoid toxin genetic islet

**DOI:** 10.1016/j.jbc.2024.107758

**Published:** 2024-09-12

**Authors:** Sarah C. Gartly, Luke A.F. Barretto, Anne-Charlotte M.T. Côté, Zach A. Kosowan, Casey C. Fowler

**Affiliations:** Department of Biological Sciences, University of Alberta, Edmonton, Alberta, Canada

**Keywords:** bacterial pathogenesis, *Salmonella enterica*, *Salmonella* Typhi, typhoid fever, bacterial toxins, typhoid toxin, phospholipases, phospholipase A2

## Abstract

*Salmonella* Typhi, the cause of typhoid fever, is a bacterial pathogen of substantial global importance. Typhoid toxin is a secreted AB-type toxin that is a key *S*. Typhi virulence factor encoded within a 5-gene genetic islet. Four genes in this islet have well-defined roles in typhoid toxin biology; however, the function of the fifth gene is unknown. Here, we investigate the function of this gene, which we name *ttaP*. We show that *ttaP* is cotranscribed with the typhoid toxin subunit *cdtB*, and we perform genomic analyses that indicate that TtaP is very highly conserved in typhoid toxin islets found in diverse salmonellae. We show that TtaP is a distant homolog of group XIV secreted phospholipase A2 (PLA2) enzymes, and experimentally demonstrate that TtaP is a *bona fide* PLA2. Sequence and structural analyses indicate that TtaP differs substantially from characterized PLA2s, and thus represents a novel class of PLA2. Secretion assays revealed that TtaP is neither cosecreted with typhoid toxin, nor is it required for toxin secretion. Although TtaP is a phospholipase that remains associated with the *S.* Typhi cell, assays that probed for altered cell envelope integrity failed to identify any differences between WT *S*. Typhi and a *ttaP* deletion strain. Collectively, this study identifies a biochemical activity for the lone uncharacterized typhoid toxin islet gene and lays the groundwork for exploring how this gene factors into *S*. Typhi pathogenesis. This study further identifies a novel class of PLA2, enzymes that have a wide range of industrial applications.

Typhoid fever, a major health issue in the economically developing world, is caused by human-adapted serotypes of the bacterial pathogen *Salmonella enterica*, most notably the Typhi serovar (*Salmonella* Typhi) (1, 2). There are marked differences in the pathogenesis of typhoidal salmonellae compared to nontyphoidal salmonellae, which are a common cause of gastroenteritis (3, 4). Despite this, the *S*. Typhi genome encodes few overt virulence factors that are absent from common broad host range serotypes such as Typhimurium and Enteritidis (5, 6). One such virulence factor is typhoid toxin, a secreted AB-type toxin that is only encoded by select lineages of the *Salmonella* genus (7, 8, 9, 10). Typhoid toxin modifies host cell biology *via* its two active (A) subunits, the ADP-ribosyltransferase PltA and the DNase CdtB. The function of PltA is not yet understood, and typhoid toxin phenotypes identified to date all stem from the DNA damage elicited by CdtB, which triggers cell cycle arrest or cell death in intoxicated cells (11, 12, 13). Typhoid toxin’s A subunits assemble into an A_2_B_5_ holotoxin featuring one copy of each A subunit in complex with a delivery (B) subunit homopentamer (13). The delivery subunits are essential for the cell binding and intracellular trafficking of typhoid toxin, which is mediated through their ability to bind sialoglycan receptors (12, 13, 14, 15, 16, 17). *S*. Typhi produces two different versions of typhoid toxin that feature distinct B subunits, PltB and PltC, and which exhibit different properties; this two-toxin arrangement appears to be a conserved feature of typhoid toxin biology in the diverse assortment of *Salmonella* lineages that encode this toxin (9, 10, 18, 19, 20). Typhoid toxin recapitulates symptoms of severe typhoid fever in animal models of intoxication and is proposed to play an important role in the systemic stages of *S*. Typhi infection, although there is a great deal that remains unknown about its activities *in vivo* (7, 13, 18, 19, 21, 22).

Typhoid toxin has an unusually complex biological program for an AB-type toxin. Expression of the typhoid toxin genes is strongly repressed until the intracellular stage of infection when *S*. Typhi inhabit a membrane-bound niche known as the *Salmonella* containing vacuole (SCV) (18, 23). Cues sensed within the SCV trigger robust expression of the toxin subunits, which are translocated to the periplasm *via* the Sec secretion system, where holotoxin assembly occurs (12, 18, 23). Typhoid toxin is then secreted out of the *S*. Typhi cell from the periplasmic space using an incompletely understood secretion mechanism that hinges on the activity of TtsA, a muramidase encoded from within the typhoid toxin genetic islet (24, 25, 26). TtsA, which exhibits specificity for L,D-crosslinked (3,3-crosslinked) peptidoglycan, remodels the cell wall to permit toxin leakage from the cell (25). *In vitro*, TtsA activity is insufficient for toxin secretion, and membrane-active agents that perturb the outer membrane (OM) must be added to enable TtsA-dependent toxin release (25). OM-perturbing agents such as antimicrobial peptides are thought to be present in the SCV and are proposed to facilitate typhoid toxin secretion during infection (25, 27, 28). Following its secretion, typhoid toxin binds the cation-independent mannose-6-phosphate receptor within the SCV, triggering its packaging into exocytic vesicles that export typhoid toxin to the extracellular space (15, 17). Extracellular typhoid toxin then binds sialoglycan receptors on target cells, leading to toxin uptake, retrograde trafficking, and ultimately cellular intoxication (12, 13, 29).

The typhoid toxin genetic islet is composed of five genes, which includes three toxin subunits (*pltB*/*pltA*/*cdtB*) and the *ttsA* secretion factor. The final gene in the typhoid toxin islet, *sty1887* in *S*. Typhi CT18, is an uncharacterized gene that encodes a putative 125 amino acid protein. Previous studies have shown that *sty1887* is dispensable for typhoid toxin’s effects in cell culture models of infection, but nothing is known about its activity or function (19, 24). In this study, we investigate this mysterious gene. We determine that *sty1887* encodes a novel phospholipase A2 (PLA_2_) and we explore its function as well as its link to typhoid toxin biology. Based on our findings, we have renamed this gene *ttaP* (typhoid toxin associated phospholipase).

## Results

### TtaP is highly conserved within the typhoid toxin islets of diverse salmonellae

**Figure 1:**
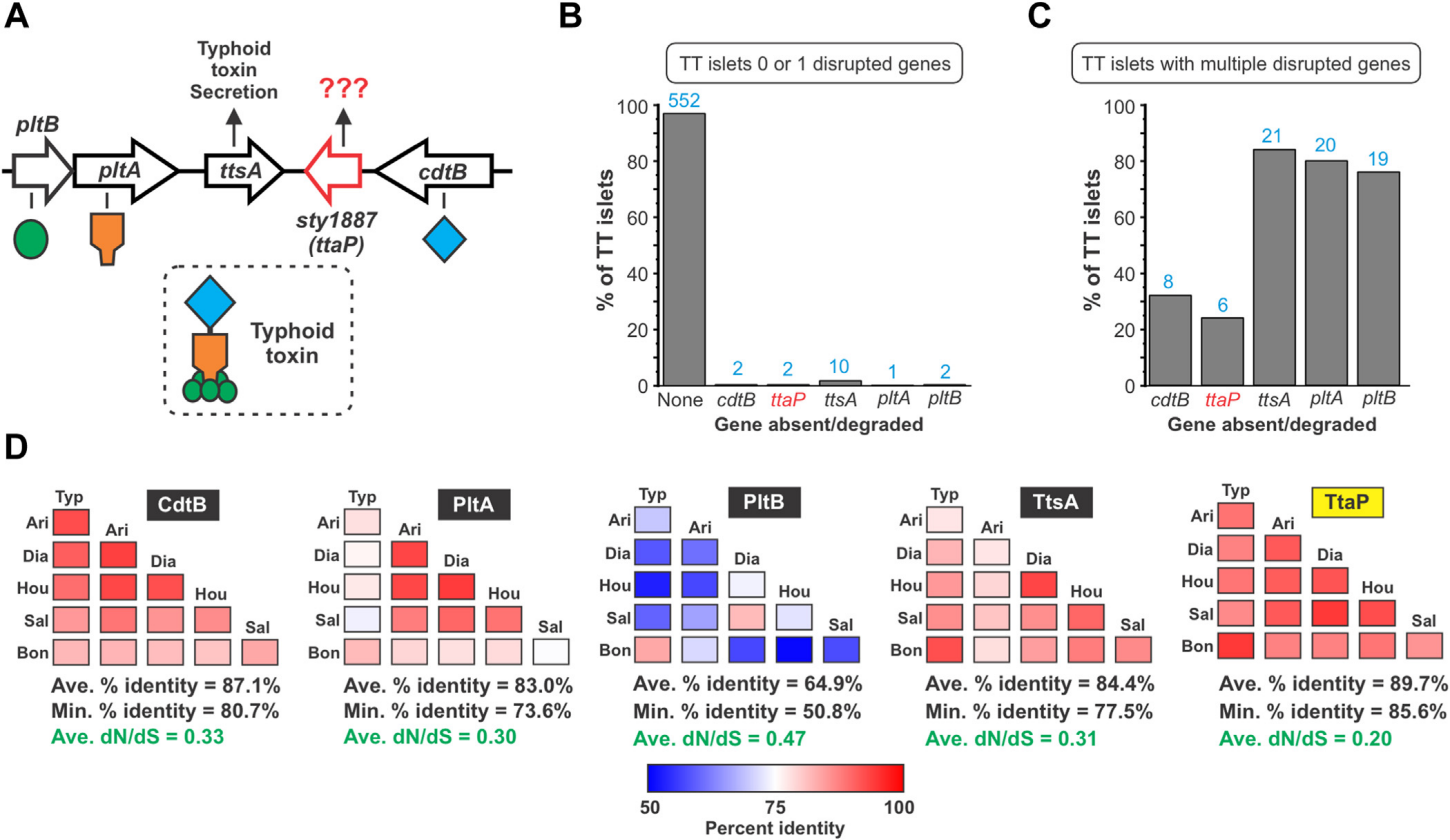
**TtaP is highly conserved across diverse typhoid toxin encoding *Salmonella* lineages.***A*, schematic representation of the typhoid toxin islet showing the functions of its five genes. *B* and *C*, the percentage of typhoid toxin islets in *Salmonella* genomes within the NCBI nr DNA sequence database in which each gene is absent or degraded. *B*, data for the 569 genomes in which all five of the typhoid toxin genes are intact, or where there is a single disrupted gene. *C*, data for the 25 genomes in which multiple typhoid toxin islet genes are disrupted. *Blue* numbers shown above the bars represent the raw number of genomes where the given gene is disrupted. *D*, percent amino acid identity matrices for each typhoid toxin islet protein across representatives from six diverse *Salmonella* lineages: Typ, *S*. Typhi str. Ty2 (*Salmonella enterica*, *enterica* subsp.); Ari, *Salmonella arizonae* str. 509917 (*S. enterica*, *arizonae* subsp.); Dia, *S. diarizonae* str. 553278 (*S. enterica*, di*arizonae* subsp.); Hou, *Salmonella houtenae* str. NCTC7318 (*S. enterica*, *houtenae* subsp.); Sal, *S. salamae* str. 2015K-0023 (*S. enterica*, *salamae* subsp.); Bon, *S. bongori* str. SARC 11 (*S. bongori* species). The six sequences for each protein were aligned using Clustal Omega and pairwise percent sequence identity values for each combination were converted to a *red-blue* color scale according to the provided legend. The average dN/dS value for each gene across all pairwise combinations of the six representative lineages is shown in *green text*. Raw data relevant for panels (*B*) and (*C*) are available as Supplemental Dataset 1. TtaP, typhoid toxin associated phospholipase.

To explore whether TtaP is likely to serve an important function, or if it is just a remnant of typhoid toxin evolution, we took a genomics approach. Horizontally-acquired genes with specialized functions are commonly found as pseudogenes in bacterial genomes (30). Typhoid toxin is thought to have ancient origins and has been extensively transferred within the *Salmonella* genus (10, 18); we therefore reasoned that if *ttaP* did not serve an important function, it would be degraded in a relatively high proportion of typhoid toxin islets. To test this, we screened the NCBI nr DNA sequence database and identified a total of 594 typhoid toxin-encoding *Salmonella* genomes (Supplemental Dataset 1). This dataset covers a broad spectrum of salmonellae; although *S*. Typhi and *S*. Paratyphi A genomes are overrepresented, ∼70% of the identified typhoid toxin islets are from other lineages, including dozens of different serovars scattered across the genus. We found that in the vast majority of genomes (552/594, ∼93%) all five typhoid toxin islet genes are intact (Fig. 1*B*, Supplemental Dataset 1). This is noteworthy, since there is substantial sequence variation among the typhoid toxin islets in our dataset, with different lineages exhibiting as many as ∼800 base pair differences over the ∼3300 bp islet (Supplemental Dataset 1). Among the 17 strains identified wherein only one of the typhoid toxin islet genes was disrupted, *ttsA* was disrupted in ten strains, while *ttaP*, *cdtB*, *pltA*, and *pltB* were each disrupted in 1 to 2 genomes (Fig. 1*B*). That the coding sequence of *ttaP* is intact across diverse typhoid toxin islets with divergent DNA sequences implies that it confers a strong fitness advantage. We reasoned that if this fitness advantage were unrelated to typhoid toxin, then the coding sequence of TtaP would be similarly conserved in genomes where other typhoid toxin genes are disrupted. However, *ttaP* was degraded in six of the 25 genomes (∼24%) in which multiple typhoid toxin islet genes were disrupted, a substantially higher rate than was observed for genomes where *pltA*, *cdtB*, *pltB*, and *ttsA* are all intact (∼0.3%) (Fig. 1*C*). These results therefore support the premise that TtaP confers a fitness advantage that is related to typhoid toxin.

We next examined the amino acid sequence conservation of typhoid toxin islet proteins. As a cross section of typhoid toxin islet diversity, we analyzed the CdtB, PltA, PltB, TtsA and TtaP sequences from six genomes in our dataset: one representative from each of the five *S. enterica* subspecies where a typhoid toxin islet was found, and one from the *Salmonella bongori* species. For each of the typhoid toxin islet proteins, we determined the percent amino acid sequence identity for each of the 15 pairwise combinations of these six strains. This analysis revealed that the PltB sequences of different typhoid toxins vary substantially in different lineages, while the sequences of the other proteins were much more highly conserved (Fig. 1*D*). These salmonellae are associated with different host species whose cells/tissues exhibit different glycosylation patterns (31, 32, 33), and this finding likely reflects the selective pressure for PltB to adapt its glycan binding properties. Importantly, TtaP’s sequence was the most highly conserved of all the typhoid toxin islet proteins, both in terms of the average and the minimum percent sequence identities across these six lineages (Fig. 1*D*). Given the DNA-level variability of the islets being compared, this high level of TtaP amino acid sequence conservation indicates that mutations to TtaP generally have a fitness cost, and also suggests that the function and the properties of TtaP are likely to be similar across different typhoid toxin encoding lineages. To further examine the evolutionary pressure on the typhoid toxin islet proteins, we analyzed the ratio of nonsynonymous to synonymous substitution rates (*dN/dS*) for all five genes across the six representative typhoid toxin islets. We found that the average *dN/dS* value for each of the genes was below 1, signifying a net negative (purifying) selection against mutations that result in an altered coding sequence (Fig. 1*D*). Of all typhoid toxin islet genes, the average *dN*/*dS* value was the lowest for *ttaP*, again signifying that *ttaP* confers a strong fitness advantage. Collectively, the results of our genetic analyses indicate that TtaP serves an important function that is likely to be somehow related to typhoid toxin biology.

### *ttaP* is cotranscribed with *cdtB via* its PhoPQ-activated promoter

**Figure 2:**
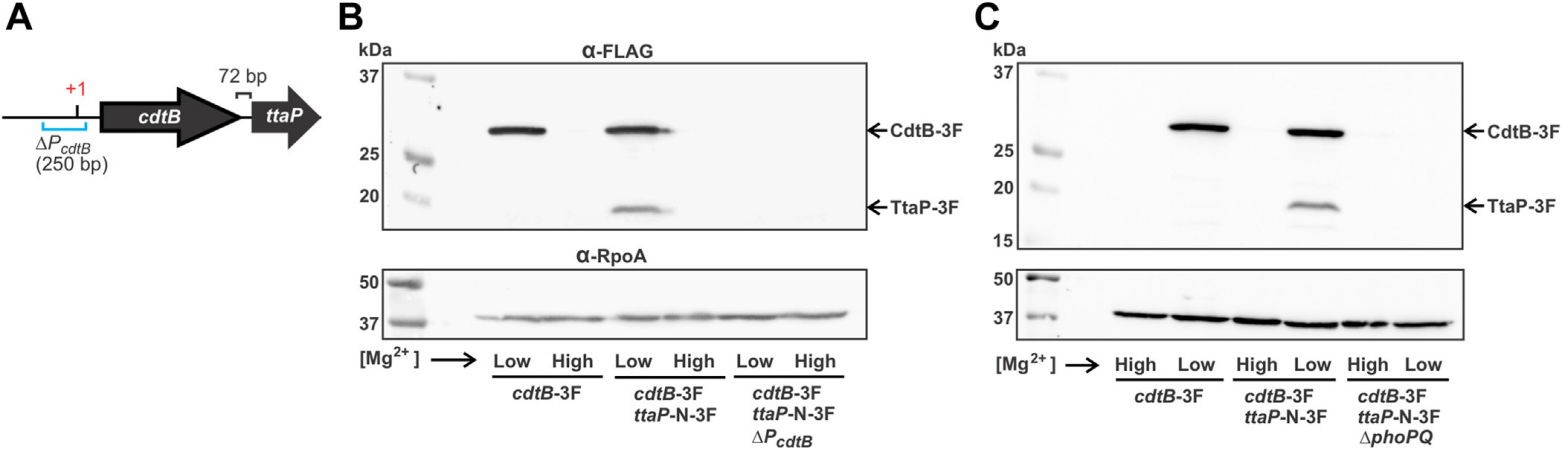
**TtaP is cotranscribed with *cdtB via* its PhoP-activated promoter.***A*, schematic of the *cdtB*/*ttaP* genomic locus. *cdtB* and *ttaP* are transcribed from the same strand and are separated by 72 bp. The transcriptional start site (+1) of the previously mapped *cdtB* promoter is shown, along with the 250 bp region deleted in the *cdtB* promoter deletion strains (ΔP_cdtB_). *B* and *C*, the indicated 3F-tagged *S.* Typhi strains were grown in TTIM containing either a low (10 μM) or a high (10 mM) concentration of Mg^2+^ for 24 h. The bacteria were then collected and whole cell lysates were analyzed by Western blot using an α-FLAG antibody, as well as an α-RpoA antibody, which served as a loading control. Each experiment was conducted independently at least twice with equivalent results. Additional data relevant for this figure can be found as Fig. S1. TtaP, typhoid toxin associated phospholipase

Given its genomic context (Fig. 1*A*), we reasoned that the expression of *ttaP* might be coregulated with the typhoid toxin genes. The typhoid toxin islet genes are expressed from two promoters, one that controls *pltB*/*pltA*/*ttsA* expression and one that controls *cdtB* expression (23). Both promoters are under tight regulatory control and are strictly repressed by the transcriptional silencer H-NS until *S*. Typhi encounters the SCV, at which point their expression is strongly induced through direct activation by the PhoP/PhoQ (PhoPQ) two-component system (23). *ttaP* is encoded ∼70 bp downstream of *cdtB* and is transcribed from the same strand, suggesting that these genes might be cotranscribed from the previously mapped *cdtB* promoter (Fig. 2*A*) (23). There is an in-frame stop codon <15 bp upstream of the annotated *ttaP* start codon, ruling out the possibility that the true start codon lies closer to *cdtB*. To explore the regulation of *ttaP*, we generated a *S*. Typhi strain carrying 3xFLAG (3F) epitope tags on both TtaP and CdtB. We then used Western blot analysis to track the levels of both proteins following growth in TTIM, a growth medium with a low Mg^2+^ concentration that activates PhoPQ, leading to high levels of typhoid toxin expression (23). An otherwise identical medium that contains a high concentration of Mg^2+^ (TTIM + Mg^2+^) and does not promote PhoPQ activity or typhoid toxin expression served as a control. We found that, like CdtB, TtaP levels were high in TTIM, but undetectable in TTIM + Mg^2+^ (Fig. 2*B*). Importantly, levels of both CdtB and TtaP were undetectable in a strain carrying a deletion to the *cdtB* promoter (ΔP_*cdtB*_, which carries a 250 bp deletion spanning from ∼200 bp upstream through ∼50 bp downstream of the previously mapped +1 site), which suggests that *cdtB* and *ttaP* are cotranscribed using the same promoter (Fig. 2*B*). These results used a strain in which TtaP carries an N-terminal 3F epitope tag (TtaP-N-3F); similar results were observed using a strain where TtaP instead has a C-terminal 3F tag (TtaP-C-3F), but the band intensity of TtaP-C-3F was somewhat lower (Fig. S1*A*). To confirm that *ttaP* and *cdtB* are cotranscribed, we grew *S*. Typhi in TTIM, isolated RNA, and performed reverse transcription PCR analysis using primers that span *cdtB*/*ttaP*, as well as a control reaction using the same *ttaP* (reverse) primer, but a forward primer that resides upstream of the P_*cdtB*_ +1 site. We found robust amplification of the test PCR, but not of the control, confirming that *cdtB* and *ttaP* are cotranscribed (Fig. S1*B*). Consistent with their coregulation *via* the established *cdtB* promoter, the levels of both CdtB-3F and TtaP-3F were also undetectable under inducing conditions in a strain carrying a Δ*phoPQ* mutation (Fig. 2*C*). Collectively, these data indicate that *ttaP* is cotranscribed with *cdtB* from its PhoPQ-inducible promoter, further implicating TtaP in typhoid toxin biology.

### *ttaP* encodes a phospholipase A2

**Figure 3:**
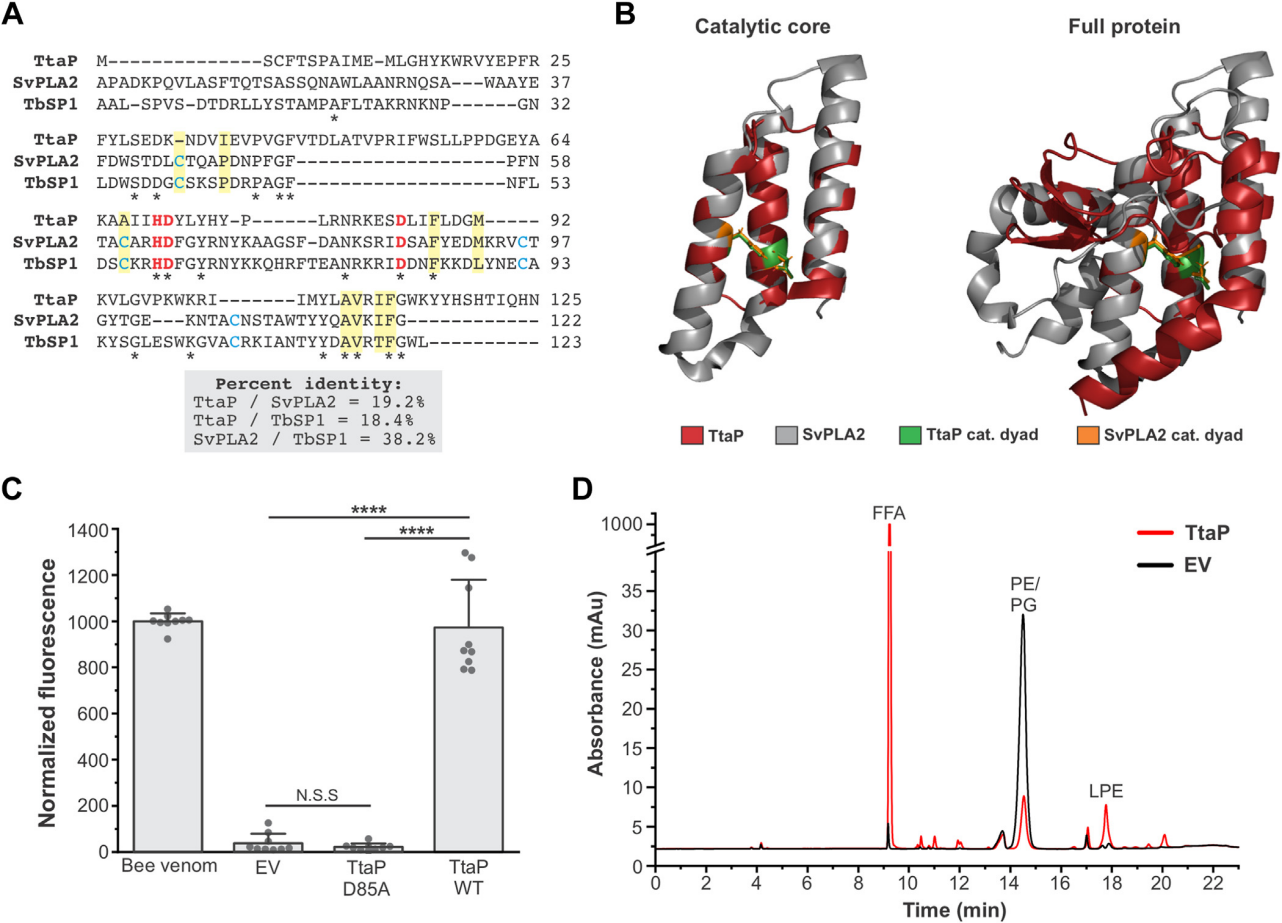
**TtaP is a novel phospholipase A2 enzyme.***A*, amino acid sequence alignment of TtaP with two biochemically verified PLA2 proteins identified as significantly similar to TtaP using HHpred searches of the PDB database. The alignment uses the mature forms SvPLA_2_ and TbSP1, both of which have N-terminal sequences that are removed during enzyme secretion (SVPLA_2_) or activation (TbSP1). Residues conserved in all three proteins are denoted with an *asterisk*. *Red* residues are conserved amino acids that play an essential role in catalysis, *yellow* highlights show residues in the putative substrate binding pocket of SvPLA_2_/TbSP1, *blue* residues are Cys that form intramolecular disulfide bonds in SvPLA_2_ and TbSP1, but are not conserved in TtaP. *B*, *ribbon diagram* showing the structure of SvPLA_2_ (PDB ID: 1LWB) overlaid with the AlphaFold-modeled structure *S*. Typhi TtaP. Two structures are shown: one that includes only the conserved catalytic core of the enzymes, and one that shows the complete protein structures. The amino acids that form the catalytic dyad for SvPLA_2_ and their TtaP counterparts are shown in *orange* and *green*, respectively, as indicated. *C*, EnzChek Phospholipase A2 Assay of lysates from *Escherichia coli* carrying either an empty vector (EV) control, a plasmid that overexpresses WT TtaP, or a plasmid that overexpressed the TtaP carrying a point mutation to the D85 residue predicted to be part of the catalytic dyad (D85A). Three biological replicates of each sample were assayed in three separate experiments (n = 9) and bars represent the average fluorescence intensity (indicative of PLA_2_ activity) normalized such that the average value of the positive control, bee venom PLA_2_, for each assay was 1000. Error bars represent standard deviation. Statistical significance of the indicated comparisons was determined by Tukey’s test: ∗∗∗∗ = *p* < 0.0001. *D*, HPLC analysis of total lipids extracted from *Escherichia coli* cell lysates overexpressing TtaP (*red* trace) and an analogous empty vector control sample (*black* trace). The labeled peaks, FFA (free fatty acids), PE/PG (phosphatidylethanolamine/phosphatidylglycerol), and LPE (lysophosphatidylethanolamine) were identified using chemical standards. These data are representative of three biological replicates for each sample. Fig. S3 shows the data for all six of these experiments, as well as peak elution times for chemical standards. PLA_2_, phospholipase A2; TtaP, typhoid toxin associated phospholipase; PDB, Protein Data Bank.

To generate hypotheses regarding the function of *ttaP*, we took a bioinformatics approach. TtaP is a 125 amino acid protein that contains a single putative domain of unknown function, DUF1353 (pfam07087), which spans the majority of the protein. To probe the function of the DUF1353 domain, we used the Conserved Domain Architecture Retrieval Tool (CDART) (34) to identify the domain architectures of proteins that contain DUF1353. This analysis identified 20 functional domains that are found within DUF1353-containing proteins (Table S1). In light of the findings described below, it is notable that this list includes two different acyltransferase domains, as well as numerous domains involved in the degradation of central biological macromolecules (a lysozyme domain, an endonuclease domain, and several different protease domains). We next performed a Hidden Markov Model (HMM) homology search for TtaP against the Protein Data Bank (PDB) database using the HHpred HMM-HMM comparison tool (35). This search revealed two high confidence hits, both of which are biochemically verified PLA_2_ enzymes. PLA_2_s catalyze the cleavage of the ester bond in the sn-2 position of phospholipids, producing a free fatty acid and a lysophospholipid that contains only one fatty acid (36). The PLA_2_s identified by HHpred are encoded by organisms from different domains; a bacterium, *Streptomyces violaceoruber* (herein dubbed SvPLA_2_, PDB ID: 1LWB), and a eukaryotic (fungal) organism, *Tuber borchii* (TbSP1, PDB ID: 4AUP) (37, 38, 39, 40). These enzymes are both members of a family designated group XIV secreted PLA_2_s, and share structural and mechanistic features, including a conserved catalytic His/Asp dyad (37, 38, 39, 40, 41). Although TtaP exhibits <20% amino acid sequence identity with SvPLA_2_/TbSP1, the critical catalytic residues are conserved in all three proteins (Fig. 3*A*) (38, 40). Specifically, the catalytic His/Asp dyad (H70 and D85 in TtaP) is conserved, as is the Asp adjacent to the catalytic His (D71 in TtaP) that is known to play an important role in catalysis. Several of the other well-conserved residues among these three proteins map to residues within the predicted phospholipid-binding pocket (Fig. 3*A*) (40). Both SvPLA_2_ and TbSP1 possess N-terminal sequences that are cleaved to produce the mature form of the enzyme. TtaP is a similar size to the processed versions of these other enzymes, and unlike the secreted bacterial enzyme SvPLA_2_, TtaP lacks an overt secretion signal sequence. An AlphaFold (https://alphafold.ebi.ac.uk/entry/Q8Z6A6)-generated structural prediction for *S*. Typhi TtaP exhibits substantial structural similarity to the experimentally determined structure of the SvPLA_2_ over the central region of the protein that contains the active site, with the catalytic residues adopting nearly identical positions and orientations within these structures (Fig. 3*B*) (42, 43). However, outside of this core three helix bundle, the predicted structure of TtaP varies from the SvPLA_2_ and TbSP1 structures, which are much more similar to one another than they are to the AlphaFold-generated TtaP structural prediction (Figs. 3*B*, S2). These predicted structural differences are consistent with the sequence alignment of the three proteins, in which there are several gaps between TtaP and the other two proteins, which align well with one another (Fig. 3*A*). Furthermore, TtaP lacks the four Cys residues that form two conserved disulfide bonds in SvPLA_2_ and TbSP1 that are thought to serve important structural roles (Fig. 3*A*) (39, 40). Collectively, these data suggested that TtaP might be a novel PLA_2_ with distinct structural characteristics compared to previously characterized PLA_2_s.

To test the hypothesis that TtaP is a PLA_2_, we first used a commercially available assay, the EnzChek Phospholipase A2 Assay Kit, in which a fluorescent signal is generated upon cleavage of the sn-2 linkage of a synthetic fluorophore-containing phospholipid substrate. We overexpressed TtaP from a plasmid in *Escherichia coli*, as well as a catalytic mutant carrying a point mutation to the predicted catalytic Asp residue (D85A), and then measured PLA_2_ activity in cell lysates (Fig. 3*C*). We found that the fluorescence level for an empty vector control was similar to the buffer only (no lysate) control, indicating that *E. coli* cell lysates lacked substantial PLA_2_ activity and did not generate a spurious fluorescent signal. Lysates from *E. coli* overexpressing WT TtaP, but not the D85A catalytic mutant, led to high levels of fluorescence that were similar to those generated by purified bee venom PLA_2_, a positive control included with the kit. These results indicate that TtaP has PLA_2_ activity. To confirm this result, we generated cell lysates from *E. coli* cells overexpressing TtaP from a plasmid, as well as from empty vector controls, carried out lipid extractions, and then analyzed the total lipid content of these samples by HPLC (Fig. 3*D*). Prior to lipid extraction, lysates were incubated at 37 °C for 30 min to allow TtaP to process phospholipids outside the context of the intact cell, where TtaP might not have access to potential substrates or where membrane homeostasis mechanisms could mask its activity. Compared to empty vector controls, samples expressing the WT version of TtaP showed a substantial drop in the levels of the predominant *E. coli* phospholipids (phosphatidylethanolamine, PE, and phosphatidylglycerol, PG) and a corresponding increase in the abundance of free fatty acids. Although less pronounced than the differences in the levels of PE/PG and free fatty acids, TtaP samples also exhibited an increased abundance of a lipid putatively identified as lysophosphatidylethanolamine based on chemical standards. These results corroborate the results of the fluorescent PLA_2_ assay and demonstrate that TtaP has phospholipase A2 activity.

### TtaP remains associated with the bacterial cell under conditions that promote typhoid toxin secretion

**Figure 4:**
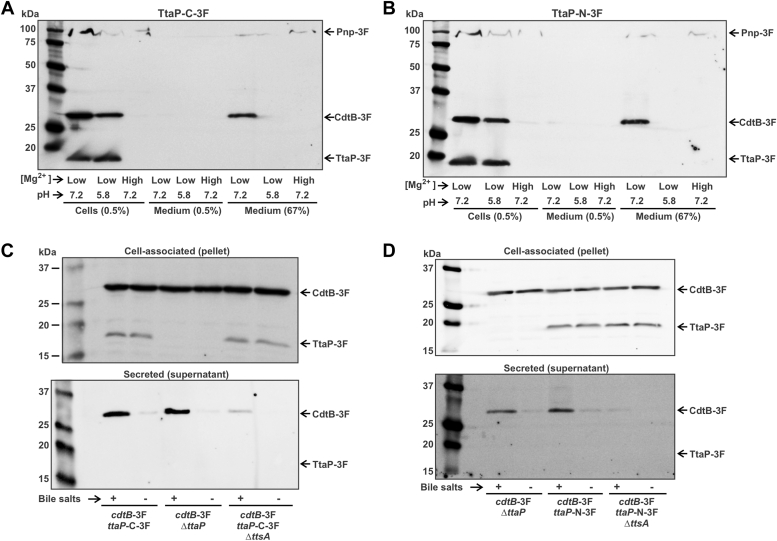
**TtaP remains associated with the *S*. Typhi cell and is not cosecreted with typhoid toxin *in vitro*.***A* and *B*, Western blot analysis of cell pellets (cell-associated material) and TCA-precipitated culture supernatants (material released into the culture medium) for *cdtB*-3F/*pnp*-3F/*ttaP*-3F *S*. Typhi following 24 h of growth in TTIM media with the indicated pH and containing either low (10uM) or high (10 mM) Mg^2+^, as indicated. Panels (*A*) and (*B*) differ only in the location of the 3F tag on the TtaP protein; C terminus for (*A*) and N terminus for (*B*). The percentage of total material from the cell or media samples that was loaded in each lane is provided at the bottom of the panel. *C* and *D*, Western blot analysis of protein secretion using the *in vitro* typhoid toxin secretion assay. The indicated strains were grown for 24 h in TTIM, concentrated, and incubated for 15 min in PBS with or without 0.05% bile salts, as indicated. Cell pellets and TCA-precipitated supernatants were then analyzed by Western blot analysis. Panels (*C*) and (*D*) differ in the location of the 3F tag on the TtaP protein; C terminus for (*C*) and N-terminus for (*D*). Each experiment was conducted independently at least twice with similar results. TCA, trichloroacetic acid; TtaP, typhoid toxin associated phospholipase.

Bacteria such as *Salmonella* are known to encode phospholipases that remain associated with the bacterial cell that are involved in processes such as maintaining membrane homeostasis, recycling membrane components, or modifying the properties of the cell envelope (44, 45, 46, 47, 48). Certain bacterial pathogens also produce secreted phospholipases that target eukaryotic host cell membranes, resulting in the disruption or modification of membrane structure and function, and potentially the generation of potent signaling molecules, such as arachidonic acid and lysophospholipids (49, 50, 51, 52). To gain insight into TtaP’s function, we therefore tested whether it is secreted or remains associated with the *S.* Typhi cell. We grew *S*. Typhi in TTIM and examined the levels of TtaP and control proteins in cell pellets (cell associated proteins) and in trichloroacetic acid (TCA)-precipitated culture medium samples (secreted proteins) by Western blot analysis. We used a strain featuring 3F epitope tags on three proteins to permit their detection: TtaP, CdtB, and the cytoplasmic enzyme polynucleotide phosphorylase (Pnp), which is not secreted and therefore its presence in the culture medium would be indicative of cell lysis. TtaP-N-3F produces a stronger signal than the TtaP-C-3F (Figs. 2*A*, S1), but because protein secretion commonly requires N-terminal signal sequences we used both strains to assess TtaP secretion. When grown in TTIM for 24 h—conditions that do not permit typhoid toxin secretion due to the absence of membrane perturbing agents—we found that TtaP, CdtB, and Pnp were all readily detected in the cell pellet, but absent from the TCA-precipitated growth medium in lanes where the same proportion of the total material was loaded (Fig. 4, *A* and *B*). To determine if low levels of these proteins were present in the culture medium, we further analyzed very dense samples of the culture media precipitate containing >100-fold more material. Here, we were able to detect CdtB and a weak band for Pnp, but could not detect TtaP. This material presumably represents a small amount of cell leakage/lysis occurring in response to prolonged growth under low Mg^2+^ growth conditions. Because the SCV where typhoid toxin is produced is acidic, we also included samples grown in a low pH version of TTIM, and also failed to detect TtaP secretion under these conditions (Fig. 4, *A* and *B*). Under all conditions, analogous results were observed for TtaP-N-3F and TtaP-C-3F, suggesting that the lack of TtaP secretion was unlikely to be due to the epitope tag.

Typhoid toxin secretion from the periplasm requires the activity of the muramidase TtsA and the presence of membrane perturbing agents such as bile salts or cationic antimicrobial peptides (25). To explore whether TtaP was cosecreted with typhoid toxin, we performed the established *in vitro* typhoid toxin secretion assay, which is similar to the experiment described above but involves an additional step where cells are exposed to bile salts, which perturbs the outer membrane, leading to TtsA-dependent typhoid toxin secretion (18, 25). We found that, although CdtB was secreted in a bile salts/TtsA-dependent manner in these experiments, both TtaP-N-3F and TtaP-C-3F were only found in the cell pellet fraction, indicating that TtaP is not cosecreted with typhoid toxin (Fig. 4, *C* and *D*). Collectively, these data indicate that TtaP is not secreted by *S.* Typhi in growth conditions that stimulate typhoid toxin expression and secretion, suggesting that the function of TtaP might relate to the modification of *S*. Typhi phospholipids.

### Exploring the effects of a Δ*ttaP* mutation under typhoid toxin inducing conditions

**Figure 5:**
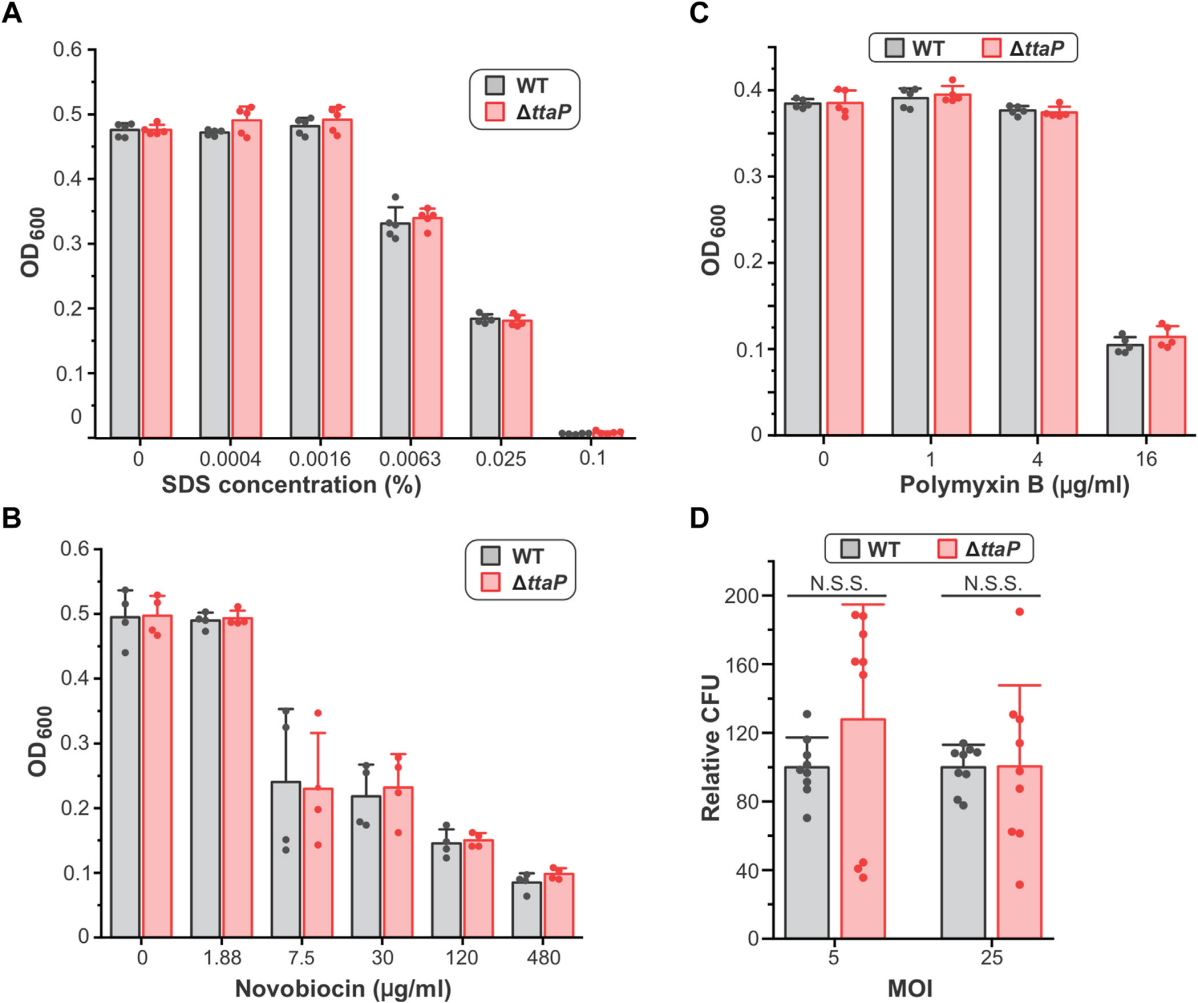
***ttaP* does not influence *S*. Typhi viability under conditions that require cell envelope integrity.***A*–*C*, WT and Δ*ttaP S*. Typhi strains were grown in TTIM for 24 h to induce *ttaP* expression, at which point they were exposed to the indicated concentrations of SDS (*A*), novobiocin (*B*) or polymyxin B (*C*) and grown for an additional 6 h (polymyxin B) or 24 h (SDS, novobiocin), at which point the absorbance (OD_600_) was measured to analyze bacterial growth. Data bars represent the average and error bars represent the standard deviation for data from two independent experiments with a total of at least four biological replicates. The growth of the WT and Δ*ttaP* strains was not significantly different under any of these conditions as determined by unpaired two-tailed t-tests. *D*, WT and Δ*ttaP S*. Typhi strains were used to infect HeLa cells at the indicated multiplicities of infection (MOIs). Gentamycin was added to the growth medium to prevent the growth of extracellular bacteria. Cells were collected and bacteria were isolated 48 h postinfection (hpi) and plated on LB-agar plates to determine the numbers of CFU recovered. Data are from three independent experiments performed in triplicate. Statistical significance was determined by unpaired two-tailed t-tests; N.S.S., not statistically significant. TtaP, typhoid toxin associated phospholipase.

Given the lack of observed secretion for TtaP, the nature of its enzymatic activity, and its association with typhoid toxin, one obvious possibility for the role of TtaP is that its membrane modifying activity might promote typhoid toxin secretion. However, previous studies have shown that Δ*ttaP* mutants are not significantly different from WT strains with respect to the levels of typhoid toxin exocytosis and intoxication in cell culture models of infection (19, 24). Consistent with this, we found that the levels of CdtB secretion in the *in vitro* typhoid toxin secretion assay were similar in WT and Δ*ttaP* strains (Fig. 4, *C* and *D*). Another related possibility is that TtaP activity might modify the properties of the cell envelope in a manner that compensates for cell envelope damage incurred through typhoid toxin’s unusual muramidase-mediated secretion mechanism. To explore this, we compared the viability of WT and Δ*ttaP* strains when exposed to chemicals known to be more potent against bacteria with compromised cells envelopes. Specifically, we assayed the sensitivity of WT and Δ*ttaP* strains to the anionic detergent SDS, the cell membrane active antibiotic polymyxin B, and to novobiocin, a large antibiotic that does not efficiently cross the OM of Gram-negative bacteria, and thus mutants with compromised OM integrity exhibit increased sensitivity to this compound (53). We found that WT and Δ*ttaP* strains did not differ in their viability when exposed to any of these agents under the conditions tested (Fig. 5, *A*–*C*). Mutations to typhoid toxin subunits do not significantly impact *S*. Typhi viability in cultured epithelial cells (23), but we reasoned that the specific environmental conditions in the SCV might necessitate TtaP-mediated cell envelope remodeling/repair, and therefore the absence of *ttaP* could impact *S*. Typhi viability during infection. We therefore assayed for *S*. Typhi growth/viability in a human epithelial cell culture model of infection using WT and Δ*ttaP* strains, and found that similar numbers of viable bacteria (colony-forming units, CFU) were recovered 48 h post infection, suggesting that the absence of *ttaP* does not significantly affect *S*. Typhi’s viability within the SCV, at least not within this cell type and on this timescale (Fig. 5*D*). Our findings do not rule out the possibility that the function of *ttaP* relates to cell envelope modifications that are important for typhoid toxin secretion or restoring homeostasis following toxin secretion, but we were unable to detect an overt phenotype in several assays that probed this possibility.

## Discussion

Typhoid toxin islets with divergent DNA sequences are found in a wide range of *Salmonella* lineages at different genome locations, suggesting this locus has ancient origins and that different alleles have been diverging for long periods of time (9, 10, 18, 19, 54). Despite this, typhoid toxin is consistently found as a five-gene locus with the coding sequence of all five genes intact. This is particularly notable given that different typhoid toxin-encoding lineages infect/colonize very different animal hosts and interact with those hosts in different ways. This suggests that all five genes make meaningful contributions to the virulence program executed by typhoid toxin. The functional relevance of the toxin subunits *cdtB*, *pltA*, *pltB*, and of the essential secretion factor *ttsA*, is clear. Here, we show that *ttaP* is conserved to an equal or greater extent as these other genes, and that *ttaP* is cotranscribed with *cdtB*. This strongly suggests that TtaP shares a functional connection to typhoid toxin, although the nature of this putative functional connection remains unknown.

Although this study did not identify TtaP’s biological function, it did unveil its biochemical activity. TtaP is a phospholipase A2, which implies that its function relates to modifying biological membranes. Our results show that TtaP remains associated with the *S*. Typhi cell under typhoid toxin/TtaP-inducing growth conditions, and that it is not cosecreted with typhoid toxin, suggesting that its function might relate to modifying *S*. Typhi membranes. Our preferred hypothesis is that TtaP’s function somehow pertains to toxin secretion, although we were unable to capture such a phenotype. TtsA’s muramidase activity is insufficient for typhoid toxin secretion, and OM perturbation is also required (25); TtaP might serve that role under conditions not captured by our *in vitro* or cell culture model systems. Lysophospholipids (generated by PLA_2_ activity) alter membrane curvature, which could promote the formation of outer membrane vesicles (OMVs) (55, 56). TtaP-mediated OMV-based delivery of typhoid toxin under some unidentified set of conditions is an intriguing possibility, but we did not observe *ttaP*-dependent OMV formation in pilot experiments that probed this possibility (data not shown). The presence of abundant lysophospholipids is disruptive to membrane integrity, and our results suggest that *ttaP* does not overtly disrupt the cell envelope when expressed by *S*. Typhi. It is therefore likely that TtaP’s activity is regulated in some way, or that it is only active against specific phospholipid substrates. Our lipidomic analysis revealed that TtaP substantially reduced the total PE/PG levels in *E. coli* lysates, and TtaP was also highly active in the EnzChek PLA_2_ assay (which uses a phosphatidylcholine-derived phospholipid substrate), which argues against TtaP exhibiting strict substrate specificity. It is therefore more likely that TtaP is spatially restricted or that it is somehow maintained in an inactive state within the *S*. Typhi cell until it is required. Determining the function of TtaP might therefore require identifying specific conditions that activate it. Our cell culture infection model showed that Δ*ttaP* mutations did not significantly change the average number of *S*. Typhi recovered from infection, but it is perhaps noteworthy that there appeared to be increased variation in the CFU numbers recovered from the Δ*ttaP* strain compared to WT between samples (Fig. 5*D*). This could indicate that TtaP is active in this infection model and is influencing *S*. Typhi biology in a meaningful way; identifying and characterizing the phenotype underlying this variation could provide an avenue to decipher TtaP’s biological function.

We did not observe TtaP secretion, but we cannot rule out the possibility that it is secreted under conditions not tested here. PLA_2_ activity can alter host cell biology in numerous ways, including by impacting the properties of membranes (*e.g.* curvature, permeability) which in turn can influence the activity of membrane-associated proteins (57, 58, 59, 60). PLA_2_s also produce free fatty acids (*e.g.* arachidonic acid) and lysophospholipids, both of which can serve as potent signaling molecules (58). TtaP’s expression under SCV-like growth conditions presents the possibility that it could be secreted by the type III secretion system (T3SS) encoded within *Salmonella* pathogenicity island 2 (SPI-2). The SPI-2 T3SS secretes numerous specific proteins (dubbed “effectors”) from within the SCV that modify host cell processes to the benefit of the bacterium (61, 62). Indeed, there are characterized T3SS effectors with phospholipase activity, including the *Salmonella* effector SseJ, which is secreted by the SPI-2 T3SS, and the prominent *Pseudomonas aeruginosa* virulence factor ExoU, which is a T3SS effector protein with PLA_2_ activity (52, 63). An argument against the possibility that TtaP is a SPI-2 T3SS effector protein, however, is that *S. bongori* also encodes TtaP (∼95% AA sequence identity to *S*. Typhi) and this species lacks the SPI-2 T3SS (10, 64). Although our results favor an activity within *S*. Typhi, it remains possible that TtaP is a distinct secreted toxin that complements the activity of typhoid toxin.

Beyond *S*. Typhi pathogenesis and typhoid toxin biology, this study also identifies a novel class of PLA_2_, with TtaP as the founding member. It is likely that other DUF1353-containing proteins also exhibit PLA_2_ activity. TtaP appears to share a distant evolutionary connection to PLA_2_ from the Group XIV family of secreted PLA_2_s found in certain symbiotic fungi and bacteria (37, 38, 41). However, sequence and structural comparisons to these enzymes reveals substantial differences, and thus TtaP is likely to have distinct properties. PLA_2_s have a wide range of industrial and biomedical applications, and TtaP has characteristics that are potentially beneficial for largescale production and purification (small size, bacterial origins, and lack of disulfide bonds) and could thus offer advantages over similar enzymes currently used for such applications (65). Further study will be required to dissect its enzymatic properties and how they compare to previously characterized phospholipases.

## Experimental procedures

### *In silico* analysis of typhoid toxin genetic islets and sequences

To identify the complete set of *Salmonella* genomes within the NCBI nr DNA sequence database that encode a typhoid toxin islet, we used a BLASTn search of this database using as a query sequence the *S*. Typhi Ty2 typhoid toxin islet sequence spanning from the start of the *cdtB* coding region through the start of the *pltB* coding region, capturing all five genes. The resulting list of hits was filtered to remove any sequences that were not from complete *Salmonella* genomes, as well as any hits that aligned over <50% of the query sequence. To confirm that this approach did not miss typhoid toxin islets with divergent sequences, we identified the hit with the lowest percent sequence identity and used its complete typhoid toxin islet sequence as the query for a second BLASTn search; this analysis revealed the same hit list, indicating that the original search efficiently captured the full spectrum of *Salmonella* typhoid toxin islets. The comprehensive nature of this list was also verified by comparisons to a more in-depth analysis of typhoid toxin diversity that we conducted recently (10). To identify genomes where genes in the typhoid toxin islet were disrupted (absent or pseudogenes) we used a two-step approach. In the first step, for each protein encoded within the typhoid toxin islet we first conducted a tBLASTn search of the NCBI nr sequence database using the amino acid sequences from *S*. Typhi strain Ty2. Hits were then filtered to remove any coding sequences that did not align over >90% of the input sequence, and to remove hits with amino acid sequence identity values below a threshold that varied protein-by-protein: CdtB (70%), TtaP (80%), TtsA (80%), PltA (65%), and PltB (50%). These thresholds represent natural break points in the data and were determined to be effective empirically by manually examining numerous hits that were above/below these thresholds to determine if they reside within a typhoid toxin islet. Using different thresholds for different proteins was necessary because (i) most of these proteins exhibit significant sequence similarity to *Salmonella* proteins that are not typhoid toxin components such as TtaP to prophage DUF1353 proteins, TtsA to prophage muramidases, or PltA to ArtA, and (ii) setting a uniformly high threshold would have excluded divergent, *bona fide* typhoid toxin proteins for less conserved proteins such as PltB. The tBLASTn results for each protein were cross referenced with the list of all typhoid toxin islets, yielding a list of genomes in which a given gene appeared to be disrupted. In the second step, each genome on this list was individually investigated to confirm that this gene was not intact. Genes were deemed to be disrupted if they were largely/completely absent, or if they contained nonsense or frameshift mutations that yielded a protein that significantly aligned over <90% of the sequence orthologous proteins.

To compare the amino acid sequence conservation of the five proteins encoded by the typhoid toxin islet, we identified six genomes within the dataset generated above to serve as a cross section of typhoid toxin sequence diversity: *S*. Typhi str. Ty2 (*S. enterica*, *enterica* subsp.), *Salmonella arizonae* str. 509917 (*S. enterica*, *arizonae* subsp.), *Salmonella diarizonae* str. 553278 (*S. enterica*, di*arizonae* subsp.), *Salmonella houtenae* str. NCTC7318 (*S. enterica*, *houtenae* subsp.), *Salmonella salamae* str. 2015K-0023 (*S. enterica*, *salamae* subsp.), and *S. bongori* str. SARC 11 (*S. bongori* species). These strains were selected based on the basis that (i) they encompass the breadth of typhoid toxin’s phylogenetic spread, covering all *Salmonella* species/subspecies where this islet can be found within our dataset, (ii) iterative BLASTn searches of assorted typhoid toxin genetic islets within our dataset indicated that these sequences cover much of the breadth of typhoid toxin sequence diversity. For each typhoid toxin islet protein, the sequences from these six strains were aligned using Clustal Omega. This alignment was used to generate percent sequence identity values for each pairwise combination. We then visualized these values using a red-blue heat map, and determined the minimum and average percent sequence identity for each protein. *dN/dS* ratios were generated using MEGA (Molecular Evolutionary Genetics Analysis) V11.0.13 software (megasoftware.net) based on DNA sequences of each typhoid toxin islet gene in the six representative typhoid toxin islets listed above. Ratios were derived for each pairwise combination of a given gene, and the average of all values for each gene is reported in Figure 1.

### Plasmids, bacterial strains, human cell lines, and culture conditions

To heterologously overexpress *ttaP* in *E. coli* cells, the complete *ttaP* coding sequence was cloned into the IPTG-inducible pET22b plasmid *via* the NdeI and XhoI restriction sites, which adds a His_6_ tag to the C terminus of the protein. Standard PCR-based mutagenesis was used to generate an otherwise identical plasmid carrying the D85A point mutant. All plasmids were confirmed by DNA sequencing.

*S.* Typhi strains were derived from the WT strain ISP2825. Mutant strains were constructed using standard recombinant DNA and allelic exchange procedures using pSB890 as the vehicle for genetic transfer and *E. coli* β-2163 Δ*nic35* as the conjugative donor strain (66, 67). For strains featuring 3xFLAG epitopes, tags were introduced at the native genomic locus to the C terminus of the indicated gene unless otherwise stated. The amino acid sequence of the 3xFLAG epitope tag was as follows: DYKDHDGDYKDHDIDYKDDDDK. For *ttsA* deletion strains, the entire coding sequence of *ttsA* was deleted (start codon to stop codon) without introducing a scar. The *cdtB* promoter deletion is described above and also is a clean deletion that did not introduce any scars.

Bacteria were generally cultured in either LB broth (routine growth or *E. coli* overexpression experiments) or in TTIM (to induce *ttaP*/typhoid toxin expression in *S*. Typhi). TTIM is a chemically defined medium based on N minimal medium (68), modified to optimize typhoid toxin gene expression in *S*. Typhi (23). TTIM (low [Mg^2+^]) contained 10 μM Mg^2+^, whereas TTIM + Mg^2+^, the equivalent high [Mg^2+^] medium, contained 10 mM Mg^2+^. Specific details of growth conditions for individual experiments are indicated where appropriate.

Cell culture experiments were performed using the human epithelial cell line HeLa (CCL-2. Validated by the supplier, American Type Culture Collection), which were cultured in Dulbecco's modified Eagle's medium (Gibco) supplemented with 10% fetal bovine serum in a 5% CO_2_ humidified incubator at 37 °C. We routinely tested this cell line for *mycoplasma* contamination using the Myco-Sniff *Mycoplasma* PCR Kit (MP Biomedicals).

### Western blot analyses

For Western blot analysis of protein levels in whole cell lysates, strains were grown as indicated, after which the *A*_600_ was measured for normalization. One milliliter of each culture was pelleted and then resuspended in ∼80 μl of 2× SDS loading buffer; the re-suspension volume of different samples was varied to normalize for differences in cell number (*A*_600_). For secretion assays, samples were prepared as described below. Samples were electrophoresed using 10 to 15% SDS-PAGE gels and transferred to nitrocellulose membranes (Bio-Rad). Membranes were blocked using 5% nonfat milk, and then incubated overnight at 4 °C with the indicated primary antibody. The primary antibodies (animal, dilution used, source, and product number) used here were the following: monoclonal α-FLAG M2 (mouse, 1:5,000, Sigma-Aldrich, F3165) and monoclonal α-RpoA (mouse, 1:5000, BioLegend, 663104). The specificity of the FLAG antibody for the 3F-tagged proteins (strains) generated was determined using strains that contained untagged versions of the protein, which in all cases no longer showed at a band at the size in question. After repeated washes, membranes were incubated with secondary antibody for two hours at room temperature covered from light using a 1:15,000 dilution of IRDye 680RD Goat anti-Mouse IgG (for FLAG/RpoB blots) from LI-COR inc, product number 926-68070. After repeated washes, membranes were visualized with a Bio-Rad ChemiDoc imaging system.

### *In silico* functional analysis of TtaP structure and domains

Domain analysis of TtaP was performed using the *S*. Typhi (strain Ty2) TtaP (t1110) using the NCBI CD-Search tool (69). The identified DUF1353 domain was then further analyzed using the NCBI Conserved Domain Architecture Retrieval Tool (CDART) (34). The vast majority of identified DUF1353-containing proteins contained no other domains (>6000 sequences in the nr database). Domains from the other identified architectures (between 3 and 28 hits each) were analyzed and listed in Table S1. To search for homologous proteins with characterized activities or functions, the HHpred HMM-HMM comparison tool (35) was used with the default settings using the *S*. Typhi TY2 TtaP sequence as the query, which was searched against the most recent PDB database available on the server as of the time the search was performed (January 2020). To compare the structure of TtaP to the structurally and biochemically characterized PLA_2_ enzymes identified by HHpred, we used the AlphaFold-generated structural prediction available *via* the AlphaFold Protein Structure Database (https://alphafold.ebi.ac.uk/entry/Q8Z6A6) (42, 43), which showed high or very high model confidence for the complete PLA2 core (the portion that aligned with SvPLA_2_). Pairwise alignments of the modeled TtaP structure, the SvPLA_2_ structure (PDB ID: 1LWB), and the TbSP1 structure (PDB ID: 4AUP) were generated using the PyMOL Molecular Graphics System, Version 2.4, Schrödinger LLC, (www.pymol.org) as follows. The indicated PDB files were imported and aligned using the default alignment settings where water molecules were removed (if included in the original PDB export) and the alignment uses all atoms between two subject proteins and performs a sequence alignment complemented with the structural superposition of regions structural similarity between the subject proteins using five cycles of refinement to reject structural outliers and identify residues with which to anchor the overall alignment.

### Analysis of TtaP PLA_2_ activity

PLA_2_ activity was analyzed using two different approaches: a commercial PLA_2_ fluorescence assay kit, and HPLC lipid analysis. For the fluorescence assay, overnight culture of *E. coli* BL21 DE3 cells carrying the indicated plasmids were subcultured 1:100 into 50 ml of fresh LB-ampicillin and grown at 37 °C for 4 h, at which point IPTG was added at a final concentration of 1 mM, and growth was resumed for an additional 4 h. Following growth, the *A*_600_ of each culture was measured and used to normalize for (minor) differences in growth between cultures, and cells were pelleted and stored at −80 °C overnight. The following day, cell pellets were thawed on ice, resuspended in 5 ml of lysis buffer (20 mM Tris pH 8.0, 150 mM NaCl, 100ug/ml DNase I, 1 mg/ml lysozyme, Roche cOmplete Protease Inhibitor Cocktail), and lysed using three passages through an EmulsiFlex-B15 High-Pressure Homogenizer. Lysate was then assayed for PLA_2_ activity according to manufacturer’s instructions by adding 20 μl of cell lysate to 100 μl reactions in 96-well plates. Following a 15-min reaction time, fluorescence values were measured using a BioTek Synergy plate reader using excitation and emission values of 485/20 nm and 528/20 nm, respectively.

For the HPLC lipid analysis experiments, cultures were grown and lysates were generated as described above for the fluorescent assay. Cell lysates were then incubated at 37 °C for 30 min, at which time total lipids were extracted using a modification of the method of Folch *et al*. (70) in the presence of 50 μg of phosphatidyl dimethylethanolamine (PDME) as an internal standard. The lipid-containing phase was removed, dried under a stream of nitrogen, resuspended in 100 μl chloroform:isooctane (1:1), and injected onto the column. Lipids were separated by HPLC on an Agilent 1100 instrument using a three-solvent gradient at 1 ml/min, on a Kinetex 4.6 x 50 mm HILIC column with 2.6 um particle size (Phenomenex) as previously described (71).

### Secretion assays

For experiments to assess secretion during growth in TTIM, overnight *S*. Typhi cultures were grown in the indicated media at 37 °C for 24 h. For each sample, 20 ml of culture per experimental sample was centrifuged at 5000g for 10 min to pellet bacteria. Pellets were resuspended in PBS and aliquots were added to 2x SDS loading buffer for Western blot analysis. The supernatant was filtered using a 0.22 μm filter to remove any remaining bacteria, and then TCA-precipitated using 10% TCA and 0.1% sodium deoxycholate (final concentrations) at 4 °C overnight. Precipitates were pelleted by centrifugation at 20,000g for 45 min at 4 °C, washed twice with 80% acetone, dried, and resuspended in 2x SDS loading buffer for Western blot analysis. Typhoid toxin secretion assays were performed as previously described (18, 25). The indicated strains were grown overnight in LB, washed using TTIM, diluted 1/20 into fresh TTIM, and grown for 24 h at 37 °C. The bacteria were then pelleted, washed thoroughly, and incubated for 15 min either in 0.05% bile salts (Sigma-Aldrich) in PBS or in PBS alone (mock treated). The bacteria were then pelleted and the supernatants were filtered using a 0.2 μm filter and TCA precipitated as described above. The abundance of 3F-tagged proteins in the pellet and supernatant fractions was then determined by Western blot analysis.

### Growth/viability assays

For assays that compared the growth/viability of *S.* Typhi in the presence SDS, polymyxin B, and novobiocin, overnight cultures were pelleted and the bacteria diluted into TTIM (10 μM Mg^2+^) and grown for 24 h, conditions that lead to high levels of typhoid toxin and TtaP in the cell. The bacteria were then pelleted and resuspended in fresh TTIM lacking Mg^2+^ and the indicated concentrations of the antimicrobial agents, and were incubated at 37 °C for the indicated amount of time, at which point *A*_600_ measurements were taken to measure bacterial cell density. Prior to conducting the final assays, appropriate time points and antimicrobial agent concentration ranges were determined empirically. For epithelial cell infection assays, 10^5^ HeLa cells/well were plated in 12-well plates, and ∼24 h later, cells were infected as follows. Overnight cultures of *S*. Typhi were diluted 1/20 into fresh LB containing 0.3 M NaCl and grown to an *A*_600_ of 0.9 and then added to the HeLa cells for 1 h in Hank's balanced salt solution at the indicated multiplicity of infection. Cells were then washed twice with Hank's balanced salt solution and incubated in culture medium containing 100 μg/ml gentamycin to kill extracellular bacteria. After 1 h, cells were washed and fresh medium was added containing 5 μg/ml gentamycin to avoid repeated cycles of reinfection. 48 h post infection, cells were released from dishes using trypsin, pelleted by centrifugation, and resuspended in 0.1% sodium deoxycholate for 5 min to lyse the HeLa cells. Bacterial cells were then pelleted, resuspended, and serially diluted for CFU determination on LB-agar plates.

## Data availability

Data are generally contained within the manuscript or the Supporting information files. Any data not contained herein are available from the authors by contacting Casey Fowler by email at cfowler@ualberta.ca.

## Supporting information

This article contains Supporting information.

## Conflict of interest

The authors declare that they have no conflicts of interest with the contents of this article.

## References

[1] Dougan G., Baker S. (2014). *Salmonella enterica* serovar typhi and the pathogenesis of typhoid fever. Annu. Rev. Microbiol..

[2] Havelaar A.H., Kirk M.D., Torgerson P.R., Gibb H.J., Hald T., Lake R.J. (2015). World health organization global estimates and regional comparisons of the burden of foodborne disease in 2010. PLoS Med..

[3] Gal-Mor O., Boyle E.C., Grassl G.A. (2014). Same species, different diseases: how and why typhoidal and non-typhoidal Salmonella enterica serovars differ. Front. Microbiol..

[4] Wang B.X., Butler D.S., Hamblin M., Monack D.M. (2023). One species, different diseases: the unique molecular mechanisms that underlie the pathogenesis of typhoidal salmonella infections. Curr. Opin. Microbiol..

[5] Sabbagh S.C., Forest C.G., Lepage C., Leclerc J.-M., Daigle F. (2010). So similar, yet so different: uncovering distinctive features in the genomes of Salmonella enterica serovars Typhimurium and Typhi: genomic comparison of S. Typhimurium and S. Typhi. FEMS Microbiol. Lett..

[6] Parkhill J., Dougan G., James K.D., Thomson N.R., Pickard D., Wain J. (2001). Complete genome sequence of a multiple drug resistant Salmonella enterica serovar Typhi CT18. Nature.

[7] Galán J.E. (2016). Typhoid toxin provides a window into typhoid fever and the biology of Salmonella Typhi. Proc. Natl. Acad. Sci. U. S. A..

[8] Fowler C.C., Chang S.-J., Gao X., Geiger T., Stack G., Galán J.E. (2017). Emerging insights into the biology of typhoid toxin. Curr. Opin. Microbiol..

[9] Gaballa A., Cheng R.A., Harrand A.S., Cohn A.R., Wiedmann M. (2021). The majority of typhoid toxin-positive Salmonella serovars encode ArtB, an alternate binding subunit. mSphere..

[10] Chemello A.J., Fowler C.C. (2024). Alternate typhoid toxin assembly evolved independently in the two Salmonella species. mBio..

[11] Haghjoo E., Galán J.E. (2004). Salmonella typhi encodes a functional cytolethal distending toxin that is delivered into host cells by a bacterial-internalization pathway. Proc. Natl. Acad. Sci. U. S. A..

[12] Spanò S., Ugalde J.E., Galán J.E. (2008). Delivery of a Salmonella Typhi exotoxin from a host intracellular compartment. Cell Host Microbe.

[13] Song J., Gao X., Galán J.E. (2013). Structure and function of the Salmonella Typhi chimaeric A(2)B(5) typhoid toxin. Nature.

[14] Deng L., Song J., Gao X., Wang J., Yu H., Chen X. (2014). Host adaptation of a bacterial toxin from the human pathogen Salmonella Typhi. Cell.

[15] Chang S.-J., Song J., Galán J.E. (2016). Receptor-mediated sorting of typhoid toxin during its export from Salmonella typhi-infected cells. Cell Host Microbe.

[16] Nguyen T., Lee S., Yang Y.-A., Ahn C., Sim J.H., Kei T.G. (2020). The role of 9-O-acetylated glycan receptor moieties in the typhoid toxin binding and intoxication. PLoS Pathog..

[17] Chang S.-J., Hsu Y.-T., Chen Y., Lin Y.-Y., Lara-Tejero M., Galan J.E. (2022). Typhoid toxin sorting and exocytic transport from Salmonella Typhi-infected cells. Elife.

[18] Fowler C.C., Stack G., Jiao X., Lara-Tejero M., Galán J.E. (2019). Alternate subunit assembly diversifies the function of a bacterial toxin. Nat. Commun..

[19] Miller R.A., Betteken M.I., Guo X., Altier C., Duhamel G.E., Wiedmann M. (2018). The typhoid toxin produced by the nontyphoidal Salmonella enterica serotype javiana is required for induction of a DNA damage response in vitro and systemic spread in vivo. mBio..

[20] Ojiakor A., Gibbs R.N., Chen Z., Gao X., Fowler C.C. (2022). The evolutionary diversification of the Salmonella artAB toxin locus. Front. Microbiol..

[21] Song J., Willinger T., Rongvaux A., Eynon E.E., Stevens S., Manz M.G. (2010). A mouse model for the human pathogen Salmonella typhi. Cell Host Microbe.

[22] Del Bel Belluz L., Guidi R., Pateras I.S., Levi L., Mihaljevic B., Rouf S.F. (2016). The typhoid toxin promotes host survival and the establishment of a persistent asymptomatic infection. PLoS Pathog..

[23] Fowler C.C., Galán J.E. (2018). Decoding a Salmonella typhi regulatory network that controls typhoid toxin expression within human cells. Cell Host Microbe.

[24] Hodak H., Galán J.E. (2013). A Salmonella Typhi homologue of bacteriophage muramidases controls typhoid toxin secretion. EMBO Rep.

[25] Geiger T., Pazos M., Lara-Tejero M., Vollmer W., Galán J.E. (2018). Peptidoglycan editing by a specific LD-transpeptidase controls the muramidase-dependent secretion of typhoid toxin. Nat. Microbiol..

[26] Geiger T., Lara-Tejero M., Xiong Y., Galán J.E. (2020). Mechanisms of substrate recognition by a typhoid toxin secretion-associated muramidase. Elife.

[27] Prost L.R., Sanowar S., Miller S.I. (2007). Salmonella sensing of anti-microbial mechanisms to promote survival within macrophages. Immunol. Rev..

[28] Rosenberger C.M., Gallo R.L., Finlay B.B. (2004). Interplay between antibacterial effectors: a macrophage antimicrobial peptide impairs intracellular *Salmonella* replication. Proc. Natl. Acad. Sci. U. S. A..

[29] Chang S.-J., Jin S.C., Jiao X., Galán J.E. (2019). Unique features in the intracellular transport of typhoid toxin revealed by a genome-wide screen. PLoS Pathog..

[30] Liu Y., Harrison P.M., Kunin V., Gerstein M. (2004). Comprehensive analysis of pseudogenes in prokaryotes: widespread gene decay and failure of putative horizontally transferred genes. Genome Biol..

[31] Ferrari R.G., Rosario D.K.A., Cunha-Neto A., Mano S.B., Figueiredo E.E.S., Conte-Junior C.A. (2019). Worldwide epidemiology of *Salmonella* serovars in animal-based foods: a meta-analysis. Appl. Environ. Microbiol..

[32] Lamas A., Miranda J.M., Regal P., Vázquez B., Franco C.M., Cepeda A. (2018). A comprehensive review of non-enterica subspecies of Salmonella enterica. Microbiol. Res..

[33] Suzuki N. (2019). Glycan diversity in the course of vertebrate evolution. Glycobiology.

[34] Geer L.Y., Domrachev M., Lipman D.J., Bryant S.H. (2002). CDART: protein homology by domain architecture. Genome Res..

[35] Zimmermann L., Stephens A., Nam S.-Z., Rau D., Kübler J., Lozajic M. (2018). A completely reimplemented MPI bioinformatics toolkit with a new HHpred server at its core. J. Mol. Biol..

[36] Burke J.E., Dennis E.A. (2009). Phospholipase A2 structure/function, mechanism, and signaling. J. Lipid Res..

[37] Soragni E., Bolchi A., Balestrini R., Gambaretto C., Percudani R., Bonfante P. (2001). A nutrient-regulated, dual localization phospholipase A2 in the symbiotic fungus Tuber borchii. EMBO J..

[38] Sugiyama M., Ohtani K., Izuhara M., Koike T., Suzuki K., Imamura S. (2002). A novel prokaryotic phospholipase A2. J. Biol. Chem..

[39] Matoba Y., Katsube Y., Sugiyama M. (2002). The crystal structure of prokaryotic phospholipase A2. J. Biol. Chem..

[40] Cavazzini D., Meschi F., Corsini R., Bolchi A., Rossi G.L., Einsle O. (2013). Autoproteolytic activation of a symbiosis-regulated truffle phospholipase A2. J. Biol. Chem..

[41] Schaloske R.H., Dennis E.A. (2006). The phospholipase A2 superfamily and its group numbering system. Biochim. Biophys. Acta..

[42] Jumper J., Evans R., Pritzel A., Green T., Figurnov M., Ronneberger O. (2021). Highly accurate protein structure prediction with AlphaFold. Nature.

[43] Varadi M., Anyango S., Deshpande M., Nair S., Natassia C., Yordanova G. (2022). AlphaFold Protein Structure Database: massively expanding the structural coverage of protein-sequence space with high-accuracy models. Nucleic Acids Res..

[44] Dalebroux Z.D., Matamouros S., Whittington D., Bishop R.E., Miller S.I. (2014). PhoPQ regulates acidic glycerophospholipid content of the Salmonella Typhimurium outer membrane. Proc. Natl. Acad. Sci. U. S. A..

[45] May K.L., Silhavy T.J. (2018). The Escherichia coli phospholipase PldA regulates outer membrane homeostasis via lipid signaling. mBio.

[46] Dekker N. (2000). Outer-membrane phospholipase A: known structure, unknown biological function: MicroReview. Mol. Microbiol..

[47] Bishop R.E., Gibbons H.S., Guina T., Trent M.S., Miller S.I., Raetz C.R.H. (2000). Transfer of palmitate from phospholipids to lipid A in outer membranes of Gram-negative bacteria. EMBO J..

[48] Hsu L., Jackowski S., Rock C.O. (1991). Isolation and characterization of Escherichia coli K-12 mutants lacking both 2-acyl-glycerophosphoethanolamine acyltransferase and acyl-acyl carrier protein synthetase activity. J. Biol. Chem.

[49] Sitkiewicz I., Stockbauer K.E., Musser J.M. (2007). Secreted bacterial phospholipase A2 enzymes: better living through phospholipolysis. Trends Microbiol..

[50] Rahman M.S., Gillespie J.J., Kaur S.J., Sears K.T., Ceraul S.M., Beier-Sexton M. (2013). Rickettsia typhi possesses phospholipase A2 enzymes that are involved in infection of host cells. PLoS Pathog..

[51] Sitkiewicz I., Nagiec M.J., Sumby P., Butler S.D., Cywes-Bentley C., Musser J.M. (2006). Emergence of a bacterial clone with enhanced virulence by acquisition of a phage encoding a secreted phospholipase A2. Proc. Natl. Acad. Sci. U. S. A..

[52] Sato H., Frank D.W., Hillard C.J., Feix J.B., Pankhaniya R.R., Moriyama K. (2003). The mechanism of action of the Pseudomonas aeruginosa-encoded type III cytotoxin, ExoU. EMBO J..

[53] Klobucar K., Jardine E., Farha M.A., MacKinnon M.R., Fragis M., Nkonge B. (2022). Genetic and chemical screening reveals targets and compounds to potentiate gram-positive antibiotics against gram-negative bacteria. ACS Infect. Dis..

[54] Den Bakker H.C., Moreno Switt A.I., Govoni G., Cummings C.A., Ranieri M.L., Degoricija L. (2011). Genome sequencing reveals diversification of virulence factor content and possible host adaptation in distinct subpopulations of Salmonella enterica. BMC Genomics.

[55] Giordano N.P., Cian M.B., Dalebroux Z.D. (2020). Outer membrane lipid secretion and the innate immune response to gram-negative bacteria. Infect Immun..

[56] Fuller N., Rand R.P. (2001). The influence of lysolipids on the spontaneous curvature and bending elasticity of phospholipid membranes. Biophys. J..

[57] Fanani M.L., Ambroggio E.E. (2023). Phospholipases and membrane curvature: what is happening at the surface?. Membranes.

[58] Dennis E.A., Cao J., Hsu Y.-H., Magrioti V., Kokotos G. (2011). Phospholipase A _2_ enzymes: physical structure, biological function, disease implication, chemical inhibition, and therapeutic intervention. Chem. Rev..

[59] Mishima K., Nakajima M., Ogihara T. (2004). Effects of lysophospholipids on membrane order of phosphatidylcholine. Colloids Surf. B: Biointerfaces.

[60] Lundbaek J.A., Andersen O.S. (1994). Lysophospholipids modulate channel function by altering the mechanical properties of lipid bilayers. J. Gen. Physiol..

[61] Figueira R., Holden D.W. (2012). Functions of the Salmonella pathogenicity island 2 (SPI-2) type III secretion system effectors. Microbiology.

[62] Jennings E., Thurston T.L.M., Holden D.W. (2017). Salmonella SPI-2 type III secretion system effectors: molecular mechanisms and physiological consequences. Cell Host. Microbe..

[63] Lossi N.S., Rolhion N., Magee A.I., Boyle C., Holden D.W. (2008). The Salmonella SPI-2 effector SseJ exhibits eukaryotic activator-dependent phospholipase A and glycerophospholipid : cholesterol acyltransferase activity. Microbiology.

[64] Fookes M., Schroeder G.N., Langridge G.C., Blondel C.J., Mammina C., Connor T.R. (2011). Salmonella bongori provides insights into the evolution of the Salmonellae. PLoS Pathog..

[65] Cerminati S., Paoletti L., Aguirre A., Peirú S., Menzella H.G., Castelli M.E. (2019). Industrial uses of phospholipases: current state and future applications. Appl. Microbiol. Biotechnol..

[66] Kaniga K., Bossio J.C., Galán J.E. (1994). The Salmonella typhimurium invasion genes invF and invG encode homologues of the AraC and PulD family of proteins. Mol. Microbiol..

[67] Demarre G., Guérout A.-M., Matsumoto-Mashimo C., Rowe-Magnus D.A., Marlière P., Mazel D. (2005). A new family of mobilizable suicide plasmids based on broad host range R388 plasmid (IncW) and RP4 plasmid (IncPα) conjugative machineries and their cognate Escherichia coli host strains. Res. Microbiol..

[68] Snavely M.D., Miller C.G., Maguire M.E. (1991). The mgtB Mg2+ transport locus of Salmonella typhimurium encodes a P-type ATPase. J. Biol. Chem..

[69] Wang J., Chitsaz F., Derbyshire M.K., Gonzales N.R., Gwadz M., Lu S. (2023). The conserved domain database in 2023. Nucleic Acids Res..

[70] Folch J., Lees M., Sloane Stanley G.H. (1957). A simple method for the isolation and purification of total lipides from animal tissues. J. Biol. Chem..

[71] Abreu S., Solgadi A., Chaminade P. (2017). Optimization of normal phase chromatographic conditions for lipid analysis and comparison of associated detection techniques. J. Chromatogr. A..

