# Supplementary material for: A novel phospholipase A2 is a core component of the typhoid toxin genetic islet
Source: J Biol Chem. 2024 Sep 12;300(10):107758. doi: 10.1016/j.jbc.2024.107758 (PMC11525133; doi:10.1016/j.jbc.2024.107758)
Supplement: Supplemental Tables S1-S2 and Figures S1-S3 [file mmc1.pdf]

Table S1: Functional domains found on the same polypeptide chain as DUF1353, as identified by NCBI CDART:

Domain name	CDD Identifier[#]	Function
ZnMc Superfamily	cl00064	Zinc-dependent metalloprotease domain
SLC5-6-like sbd Superfamily	cl00456	Solute binding domain found in plasma membrane transport proteins
DUF4376	cl16786	~100 amino acid domain of unknown function
Acyltransferase 3 Superfamily	cl21495	Acyltransferase domain found in diverse enzymes with this activity
NLPC P60 Superfamily	cl21534	Domain of unknown function found in numerous lipoproteins. Members of this superfamily include cell wall hydrolases and the LRAT acyltransferases, amongst other enzymes.
COG5526 Superfamily	cl02334:	Lysozyme-family protein domain
Tryp_SPc Superfamily	cl21584	Trypsin-like serine protease domain
EndA Superfamily	cl43676	Endonuclease I domain
eMpr Superfamily	cl42390	Endopeptidase domain
PksD Superfamily	cl43841	Acyltransferase domain in polyketide synthase enzymes
Glycosyltransferase_GTB-type Superfamily	cl10013	Glycosyltransferase domain
OmpA_C-like Superfamily	cl30079	Peptidoglycan interaction domain
M34 peptidase Superfamily	cl40422	Peptidase domain
Caudo TAP Superfamiily	cl17077	Domain associated with phage tail fiber assembly proteins
YdgH BhsA-like Superfamily	cl11507	Small domain of unknown function
Peptidase S41 Superfamily	cl02526	Peptidase domain
MliC Superfamily	cl46353	Lysozyme inhibitor domain
Emfourin Superfamily	cl48409	Protealysin inhibitor domain
NapH Superfamily	cl33844	Domain found in protein involved in energy production and conversion
PutP Superfamily	cl43200	Domain affiliated with amino acid transporters

Unique identifier in the NCBI Conserved Domain Database (CDD)

Table S2: List of bacterial strains and plasmids used in this study

Strain/plasmid	Relevant features or genotype	Reference
**Strains**		
E. coli BL21 (DE3)	Widely used strain for over-expression/purification of proteins using the pET system.	PMID: 3537305
ISP2825	Wild type *S.* Typhi	PMID: 1879916
SB1946	ISP2825: *cdtB*-3xFLAG	PMID: 22042847
CCF0145	ISP2825: *cdtB*-3xFLAG, *ttaP*-N-3xFLAG	This study
CCF0036	ISP2825: *cdtB*-3xFLAG, *ttaP*-C-3xFLAG	This study
CCF0147	ISP2825: *cdtB*-3xFLAG, *ttaP*-N-3xFLAG, Δ*cdtB* promoter	This study
CCF0050	ISP2825: *cdtB*-3xFLAG, *ttaP*-C-3xFLAG, Δ*cdtB* promoter	This study
CCF0139	ISP2825: *cdtB*-3xFLAG, *ttaP*-N-3xFLAG, Δ*phoPQ*	This study
CCF0180	ISP2825: *cdtB*-3xFLAG, *ttaP*-N-3xFLAG, *pnp*-3xFLAG	This study
CCF0051	ISP2825: *cdtB*-3xFLAG, *ttaP*-C-3xFLAG, *pnp*-3xFLAG	This study
CCF0037	ISP2825: *cdtB*-3xFLAG, Δ*ttaP*	This study
CCF0053	ISP2825: *cdtB*-3xFLAG, *ttaP*-C-3xFLAG, Δ*ttsA*	This study
CCF0146	ISP2825: *cdtB*-3xFLAG, *ttaP*-N-3xFLAG, Δ*ttsA*	This study
CCF0140	ISP2825: Δ*ttaP*	This study
**Plasmids**		
pET22b (empty vector)	Vector commonly used for protein overexpression/purification	Novagen
CCF0010	pET22b + *ttaP* (WT)	This study
CCF0031	pET22b + *ttaP* (D85A)	This study

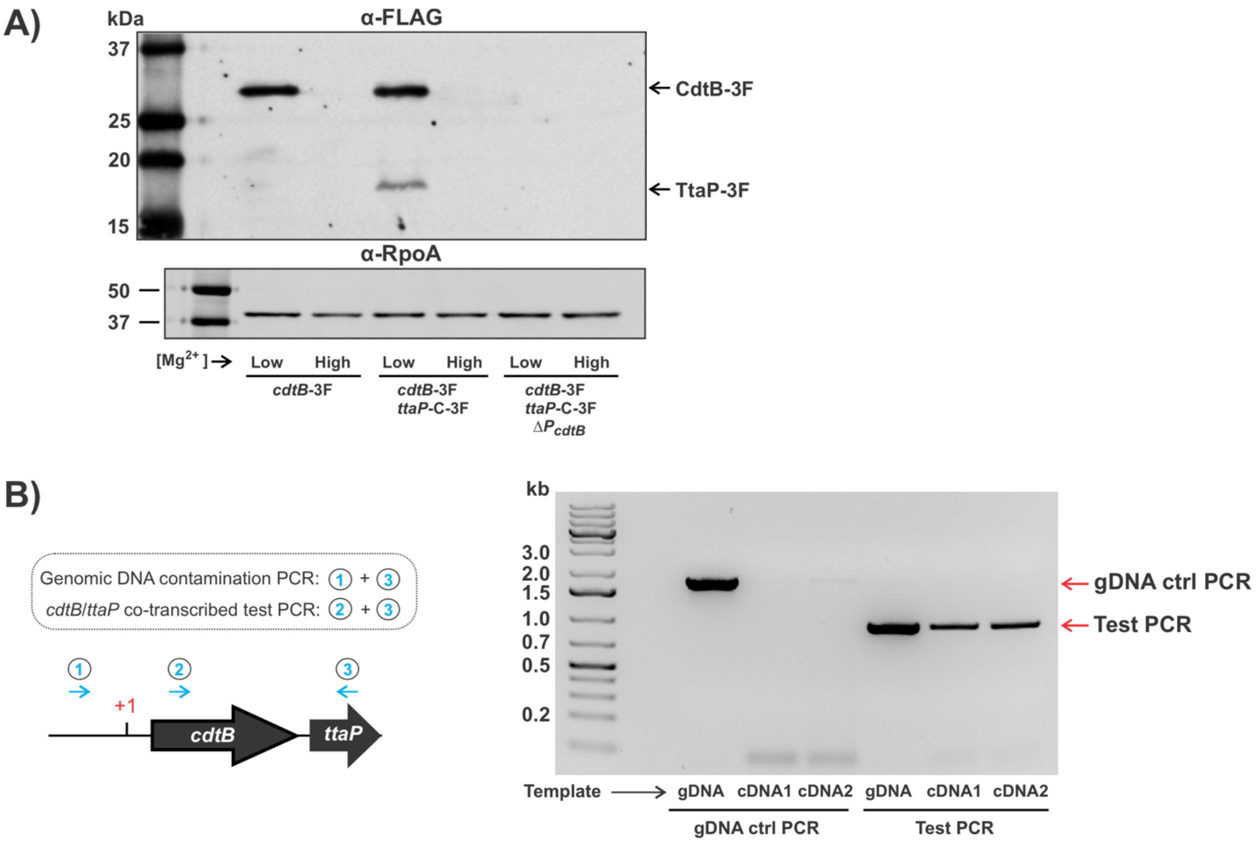

Fig. S1: *ttaP* is co-transcribed with *cdtB*. (A) TtaP-C-3F levels are only detectable under typhoid toxin inducing conditions. The indicated 3F-tagged *S.* Typhi strains were grown in TTIM containing either a low (10 μM) or a high (10 mM) concentration of Mg^{2+} for 24 hours. The bacteria were then collected and whole cell lysates were analyzed by western blot using an α-FLAG antibody, as well as an α-RpoA antibody, which served as a loading control. This experiment was conducted independently two times with equivalent results. Experiment is equivalent to Fig 2B, but uses a strain with the 3F tag on the C-terminus of TtaP rather than the N-terminus. **(B)** RT-PCR to determine whether *cdtB* and *ttaP* are co-transcribed. *S*. Typhi was grown in TTIM (10 μM Mg^{2+}) and RNA was extracted, DNase-treated, and reverse transcribed to produce cDNA. PCR using the primer sets indicated in the graphic were then used to determine if *cdtB* and *ttaP* are co-transcribed (*cdtB* and *ttaP* internal primers, labelled 2/3, expected size 937 bp) and to test for genomic DNA contamination of the cDNA samples (primer that resides upstream of the +1 site for the *cdtB* promoter and *ttaP* internal primer, labelled 1/3, expected size 1761 bp). PCR reactions were run on a 1% agarose gel and DNA was visualized using ethidium bromide. Two independent RNA isolations were tested (cDNA1 and cDNA2), and purified genomic DNA (gDNA) served as a control.

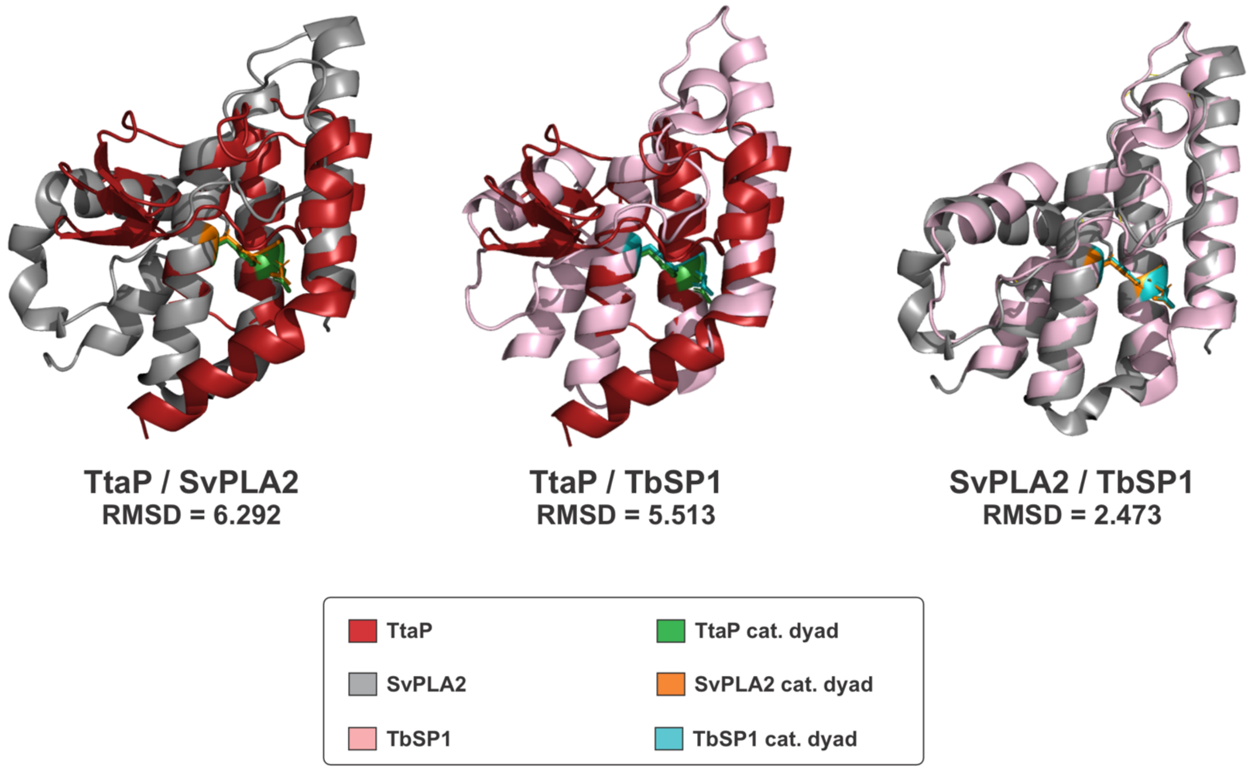

Fig. S2: Pairwise structural alignments of TtaP and related PLA_2 enzymes. Ribbon diagrams showing pairwise overlaid structures of $SvPLA_2$ (PDB ID: 1LWB), TbSP1 (PDB ID: 4AUP) and the AlphaFold modelled structure *S*. Typhi TtaP. Structures shown represent the complete mature versions of the proteins. The amino acids that form the catalytic dyad are coloured as indicated. RMSD values for each structural comparison are shown, which were calculated based on the alpha carbons across the full protein sequences.

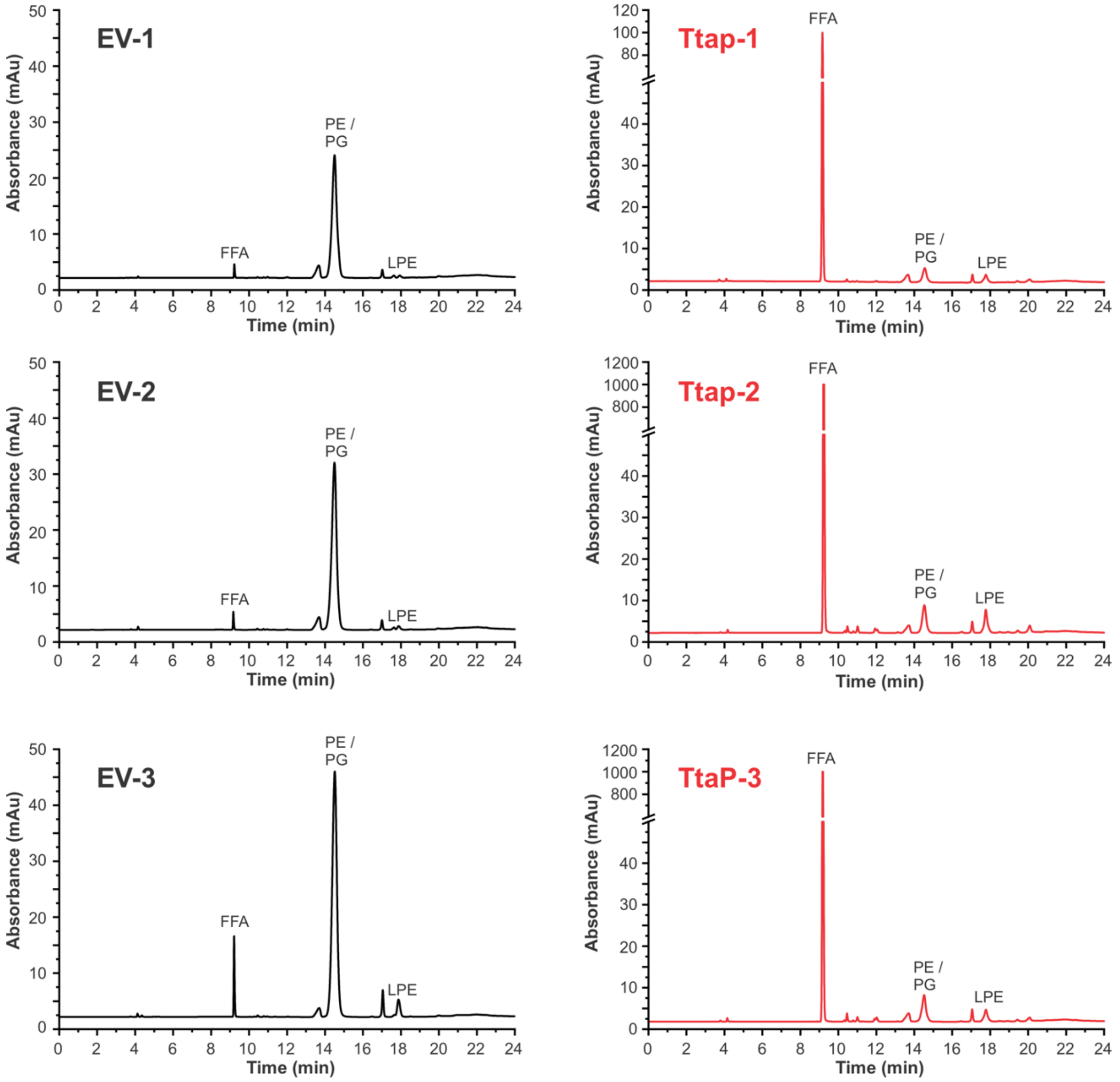

Standard	Time
FFA	9.2
PE	14.3
PG	14.5
PDME	17.0
LPE	17.6
CL	18.5

Fig. S3: HPLC analysis of lipids from *E. coli* lysates comparing cells overexpressing TtaP to empty vectors controls. HPLC analysis of total lipids extracted from of *E. coli* cell lysates, incubated at 37°C for 30 minutes to promote TtaP processing of phospholipids in cell lysates. Samples over-expressing TtaP (red traces) and analogous empty vector control samples (black traces) are shown. Samples EV-2 and TtaP-2 are shown as representative traces in Fig 3D. The labelled peaks, FFA (free fatty acids), PE/PG (phosphatidylethanolmine/ phosphatidylglycerol), and LPE (lysophosphatidylethanolamine) were identified using chemical standards. The peak elution times of relevant chemical standards are shown (box, lower left); CL (cardiolipin), PDME (phosphatidyldimethylethanolamine, used as an internal standard). The peaks for PE and PG overlap, and thus this peak encompasses both of these phospholipids.
